# World Wide Web for Crystallography

**DOI:** 10.6028/jres.101.040

**Published:** 1996

**Authors:** H. D. Flack

**Affiliations:** Laboratoire de Cristallographie, University of Geneva, Switzerland

**Keywords:** conference, crystallography, distance teaching, e-mail, publishing, Virtual Library, World Wide Web

## Abstract

Some characteristics of the World Wide Web (WWW) and its Virtual Library (W3VL) are described. Aspects of the setting up, maintenance, future development and objectives of the *World Wide Web Virtual Library: Crystallography* are detailed. An overview of the successful use of WWW in the organisation of two crystallographic conferences and one entirely electronic conference is given. A revolution in scientific publication is under way with the introduction of WWW and CD-ROM technologies and a few of the points important to crystallography are touched upon. An application to distance teaching in crystallography is described. There is no mention of WWW applications to crystallographic databases in this paper as others at the Workshop have adequately described their work.

## 1. The World Wide Web

The WWW [1] is an Internet-based distributed hypermedia system developed by T. Berners-Lee whilst working at CERN. As such its originality lies in the combination of hypertext with the Internet computer network. This results in a seamless view of information from the four corners of the world that is available at the click of a mouse. Further, although the WWW has its own native transfer protocol HTTP [2] and file format HTML [3], Berners-Lee thought that it was essential for the WWW to be compatible with the other major transfer protocols existing on the Internet. In this way, he was led to the invention of the URL (uniform resource locator) [4] as a general way of expressing locations and protocols. The HTML markup language was designed to indicate the logical and semantic context of a document rather than its physical appearance as print on paper or pixels on a screen. The form in which a web document appears on the user’s screen is a problem that has to be resolved by the particular browser (client) software depending on the hardware available and user preferences. Clearly more can be achieved on a top-of-the-range graphical workstation than on a basic alphanumeric terminal. For a crystallographer wishing for a beginner’s introduction to the WWW, I would strongly recommend a recent article by Winter, Rzepa, and Whitaker [5] written particularly with the needs of chemists in mind.

Taking one step back from the WWW, it is of use to reflect on some of the characteristics specific to its underlying layer, the Internet, and the way that these two systems are related and interact one with another. Very briefly, Internet was conceived as a bottom-up technology fundamentally rooted in extremely open and accessible standards, contrasting sharply in this respect, for example, with the telephone systems used around the world. Standards are arrived at by an open system of consensus without voting from anyone wishing to participate. The HTTP and HTML standards for WWW were also made open and accessible and even some very important recent developments by commercial companies have been made open and accessible. Unlike the telephone system, tariffs on the Internet are not based on distance but on connection, and this has given rise to the phenomenon of *The Death of Distance*. Until recently the Internet was only known to the academic and research community when the advent of the WWW itself abruptly pushed it into the public eye through its great potential for commerce. Nevertheless Internet connectivity in the world is small and limited to particular sectors of community. World wide there are 100 times more telephones than Internet connections. A recent forum on the Internet [6] may be consulted for a wealth of interesting information.

The WWW technology enables a computer-literate individual with minimal resources to become a publisher, thus communicating his thoughts, science, art, music or technology to anyone anywhere in the world. The basics of HTML can be learned in less than 60 minutes and one only needs a rudimentary text editor as a tool. Institutions, associations and commercial enterprises have not been slow to capitalize on the immense potential of this system, leading to novice users frequently being overwhelmed by the vast supply of information now available. The WWW has even been described as being akin to the Library of Congress with all of the books heaped up on the floor and the lights switched out. In part this is due to many information providers being inexperienced in the use of distributed hypertext and probably having not read Berners-Lee’s excellent counsel [7] on style. In part it is due to a phenomenon known as “shovelware” in which documents prepared for distribution as printed paper are simply copied onto the WWW without further ado.

## 2. W3VL: Crystallography^1^

Berners-Lee originated the World Wide Web Virtual Library, (W3VL) [8], to create a global, distributed and authoritative resource structuring the information available over the WWW. The work force necessary to accomplish this task is drawn up on a voluntary basis from people knowledgeable in a particular subject area or of a particular geographic or national region. In true WWW style, W3VL was designed as a distributed system, each site operating its own WWW server. A certain style in the formatting of the individual components of the W3VL was requested to create a unified presentation. The W3VL main server provides both the administrative organisation and a central point for lists of hyperlinks to the individual subject and regional servers. In turn the latter provide global indexes of WWW servers relevant to their subject matter. The content of the individual contributions to the W3VL varies enormously from one subject area to another, this being due essentially to human rather than technical factors. At one extreme there are W3VL sites providing no more than a single list of relevant servers. At the other, the editor has created a virtual encyclopaedia of his subject area.

*W3VL: Crystallography* [9] was created by Flack (1994) [10] following experience with the European *CONCISE* information server and the *Crystallography in Europe* WWW server. The usage is truly world wide and the most frequently consulted sections are those dealing with employment, software, meetings and, rather surprisingly, the editor’s personal details. The server in its present state offers very little information in the form of bitmap graphics, provides no server-side processing through the common gateway interface (cgi-bin) protocol, and has all information distributed from one single server. Each page has visual elements allowing its immediate identification as belonging to the *W3VL: Crystallography*. These are two clickable icons at the top of each page, completed at the bottom by a characteristic signature and acknowledgment. It has to be admitted that a fair amount of experimentation was necessary to come to the current arrangement for the layout and content of the indexes some of which clearly need complete redesigning and extending.

An essential advantage of the WWW over a centralised system like *CONCISE* is in its distributed nature. The evolution of *W3VL: Crystallography* indicates that an increasing proportion of information providers are now turning this fact to good use. Initially much of the information was received either as printed paper, necessitating rekeying, or as text files by e-mail subsequently distributed from Geneva. This method makes updating laborious and slow. Increasingly, WWW or ftp servers are being set up with the result that control and updating of the information are left entirely in the hands of the local provider and the *W3VL: Crystallography* needs only to provide hyperlinks from well-arranged indexes.

For submission of information to *W3VL: Crystallography* a complementary approach in conjunction with the usenet newsgroups *sci.techniques.xtallography*, originated by Cranswick [11], and *bionet.xtallography* has been found most satisfactory. As contributors post their own articles directly to the newsgroups a wide, public, rapid and efficient distribution is assured under the author’s own signature. Postings suitable for *W3VL: Crystallography* can then be extracted, indexed and marked up by its editor. Newsgroups have the advantage of simplicity in posting and immediacy but are very unstructured and unedited. WWW has a strong advantage in the structured, edited and modifiable nature of the information that it can provide but has weaknesses for indicating where changes have occurred. Both certainly have the distinct advantage over mailing lists of only delivering items of information chosen by the user according to a title or short description.

## 3. Scientific Conferences

Two crystallographic conferences, *Aperiodic ‘94* [12] and *ACA ‘95* [13], have made use of the WWW for the distribution of organisational and programme information. In both cases, author and subject indexes, and the complete texts of the abstract of each contribution were put on offer. Some details of the methods used are given by Flack [10] and Le Page, Rodgers, and Potter [14]. Extensive coverage of the 17th IUCr Congress and General Assembly, Seattle, August 1996 [15] will also be made available over the WWW.

Previewing of the timetable and abstracts by participants prior to arrival at a conference site allows more to be obtained from attendance at a meeting. In the organisational stages of the conference, all programme committee members can have ready access to all texts on which critical choices are made. For a conference where these members are drawn from across a continent or the world, it is thus possible even for those furthest away to make their full contribution. For *ACA ‘95* a survey of intending participants was conducted to determine interest in the different parts of the programme. The information was used to allocate oral sessions to suitably dimensioned rooms, and to set up a timetable which minimised the inconveniences inherent in parallel sessions.

For electronic delivery of conference material to become commonplace, it is clear that the transformation of documents into both paper and web format should be as efficient as possible. Rekeying from a printed page is time-consuming and expensive. Moreover, it is a common experience that scanning short printed documents of variable quality is even less efficient than typing. So a very high proportion of contributions need to be submitted electronically. Moreover they must be in a format that is easily and naturally generated by the participant, capable of transparent electronic transmission and readily usable by the conference organiser. It is clearly essential that many of the potential participants in a conference should be accustomed to regularly using those electronic tools capable of fulfilling the above requirements.

Whole scientific conferences have already been held electronically but not as yet in the field of crystallography, although opportunities for innovation abound. For *ECTOC, Electronic Conference on Trends in Organic Chemistry*, June–July 1995 [16] about 100 000 documents were accessed in just two weeks. The conference was advertised in March 1995 and 80 abstracts were received by the end of April. These were refereed online by the panel of conference organisers and full versions of the accepted papers and posters became available at the beginning of June. Papers were open for discussion between June 12 and July 7 and participants were able to e-mail chemical structures with their contributions. Papers were of high quality and the e-mail discussions were of wide scope.

## 4. Scientific Publishing

Primary scientific journals are already being distributed over the Internet for use with either proprietary browser software or WWW interfaces. Other scientific journals and books are being offered in hypermedia form on CD-ROM. Electronic-based systems hold out the potential for far greater interactivity in their use than is possible with printed paper. Net-based systems offer very rapid delivery of prepared articles.

A recent public electronic discussion initiated by Fanwick [17] in the *sci.techniques.xtallography* newsgroup captures well the expectations and anxieties of the user community with regard to the publication of crystal structure determination results over the WWW. The questions which are raised attempt to clarify under what conditions WWW distribution should be considered as publication or not. Authors wish for rapid publication of their results but are not prepared to squander their right to recognition of original and careful work by unprotected distribution of shoddily presented documents. No matter how a scientific paper is distributed, the system of refereeing by peer review is a key element of the process that needs to be maintained throughout any technology changes. Although the primary purpose of a scientific paper is in the communication of original results, the publication also acts as a proof of the professional competence of its authors and is thus of prime importance in their employment potential.

As an example of how hypertext can increase the usefulness and attractiveness of a scientific reference work, a report on the use of statistics in crystallography can be consulted [18]. This hypertext document is the combination of two papers published by Schwarzenbach et al. (1989) [19] and Schwarzenbach et al. (1995) [20]. Although this particular document is distributed by the WWW, it is in fact in its hypertext nature rather than in its rapid distribution that it gains over the printed version. It would thus be more suited as part of a document distributed on CD-ROM. For the electronic publication on CD-ROM of large reference works to be successful, particular attention has to be paid to the design of the hypertext indexes as it is these that offer an ease of use that is difficult to rival with the printed page.

Scholarly works in any subject area need to quote their sources and crystallographers are well familiar with the system of referencing used in scientific papers. In an abstract sense the *journal-year-volume-page* (hereafter called a *Name Reference*) enables one to “find” the reference although it does not tell in which city, in which building, on which floor, at what time, on which shelf and which particular bound volume (hereafter called a *Locator Reference*). In any case, there are multiple mappings from name to locator references and the latter change over the years. With electronic publication, the referencing system is less well developed but hardly any different. An excellent system for electronic locator references has been developed, viz, the URL (Uniform Resource Locator) but one can hardly expect URLs to be more stable with time than physical locator references. Participants in the WWW have collaborated to produce more stable referencing systems of the name type which are called URNs (Uniform Resource Name) and URCs (Uniform Resource Citation) as explained by Berners-Lee [4]. Such systems have not yet evolved to the point of being suitable for regular use. Participation from the crystallographic community in the discussions concerning URNs and URCs would ensure that its needs were effectively covered.

## 5. Distance Teaching

A university-level course called *The Principles of Protein Structure* [21] has been organized making use of the WWW as its principal interface. 250 students and consultants were drawn from around the world. 30 experts in protein structure contributed graphical and hyper-textual material for the course as well as engaging the students in technical discussions via e-mail.

BioMOO was also used as a powerful means of communication on this course. This “virtual classroom” is a serious application of the gamester’s “multi-user dungeon” where several participants (students and consultants) may be simultaneously logged on to the same remote computer and can effectively “talk” to each other from their keyboards. A development of this technique into a 3D virtual chat room can be expected in the future in conjunction with virtual reality modeling systems.

## 6. Graphics and Mathematics

WWW users are only too aware that the transmission of two-dimensional bit-map colour graphics is clogging up the Internet. Although with the generalised introduction of fibre optic cables, ATM net technology and 10 Mbit/s modems attached to bidirectional TV cables one can expect throughput to increase considerably, colour bit-map graphics nevertheless remains a technique inspired from the printed page which badly utilises the display and interactive potential of electronic systems. Take for example the representation of a molecule or a crystal structure. The underlying information is taken from a connectivity table or a list of atomic coordinates. The resulting bit-map graphic occupies orders of magnitudes more storage space and takes a correspondingly longer time to transfer. Moreover the picture is static (noninteractive) and information has been lost in this process. Various approaches at various stages of development holding out the promise of delivering more powerful graphics more rapidly over the WWW are briefly described in the following list.

- Basic numerical data (e.g., connectivity or coordinates) are provided in a standardised form on the server and interpreted by specialised software activated as an external viewer through the client’s browser. Presentation style and interactivity are conditioned by the client side software.
- Basic data are provided as an object (i.e., numeric data with associated code in an object-oriented language similar to C++) on the server. On the client side, a WWW browser having the capability of interpreting the objects is used. The presentation and interactivity is limited by the code in the object and software specific to a particular domain of activity is not required.
- Basic data are marked up in a 3D virtual reality modelling language. On the client side, a browser capable of interpreting this language is necessary in general coupled with high hardware capability.

The situation with respect to mathematical formulae is similar to that of graphics. People from the printing world see these as graphs (lines on paper), mathematicians as subtle relationships among variables. Most fortunately mark up in HTML 3 (and hopefully documents marked up in SGML using other DTDs) is semantically precise, allowing it to be easily translated into other formats such as those used by mathematical software packages capable of analytical (rather than numerical) manipulations.

## 7. E-Mail

WWW is in some respects akin to a broadcast system such as radio or television. For person-to-person communication, e-mail has become very useful and popular. The e-mail system currently operating across the Internet is one that caters only for the transfer of texts of limited length written with the alphabet as used in English (i.e., with no accents) and containing lines no longer than 80 characters. Although this simple system is very good, an increase in its functionality would be to the benefit of the scientific community. Amongst the features sought for one might mention: use of accented characters and non-Roman alphabets, no limits on line length or document size, transfer of graphics, binary code and other structured documents. A way to achieve this within the existing Internet mail transfer system has been proposed by Borenstein and Freed [22] and is called MIME (Multipurpose Internet Mail Enclosure). MIME-compatible e-mail programmes, known as UAs (User Agents), are now available for all major platforms as freeware, shareware or commercial software. MIME standards for use in chemistry and molecular science have already been proposed by Rzepa, Murray-Rust, and Whitaker [23] and working applications where chemical diagrams are transferred by e-mail have been described by Winter, Rzepa, and Whitaker [5].

## 8. Financing the WWW

Replacing the distribution of information on printed paper with that by electronic means does not magically make costs diminish. Printing and mail distribution costs may disappear but will be replaced by the fixed and variable costs associated with electronic distribution. In many cases of established information sources (e.g., scientific journals) it will not be acceptable to a significant proportion of customers for the printed version to be stopped at short or even medium notice. So the information provider has to run a dual print/electronic system leading to an increase in production costs spanning several or many years. Frequently customers misunderstand the nature of the costs leading to the price of a product. Certainly one sees the cost price of computers diminish whilst their power increases. In the USA the price of telecommunications has fallen sharply since the introduction of a market-driven monopoly-free industry whereas in other parts of the world telecommunication prices are held exorbitantly high, in some places 70 times more than current United States prices. Internet-based service providers and consumer groups are lobbying for reductions and certainly the widespread use of the Internet for commerce will not be without its effect on telecommunication tariffs. Commerce over the Internet has also spurred the development of safe and reliable digital payment and money systems and a variety of these will soon be in common use.

Nevertheless an underlying business reality is that providing information of any sort on the WWW is a value-added service for which the technological costs (e.g., telecommunications, computer equipment) tend to be a small part. The expertise of the information provider or editor in discovering or generating suitable, attractive and informative documents and indexing them adequately are costly skills on which the success of the information source will depend. This is also the case for printed documents and leads to similar fixed costs in electronic distribution. There is no reason to believe that the well-established procedures for financing printed documents (viz, advertisements, government sources, subscriptions, royalties, free publicity, sale, etc.) will not be applied to WWW documents. That documents in WWW or CD-ROM form are now distributed at below cost price is a necessary ploy to accustom users to a new technology and gently wean them off a dependence on the printed page.

## 9. WWW for Which World?

For which world is the World Wide Web made and accessible? At first sight it would seem to be a typical high-technology product for the benefit of highly developed nations. Although for developing countries the situation is currently poor, the prospects are really not that gloomy. In 1995 the World Bank announced that it will start lending money to developing countries for investment in telecommunication infrastructure, this being a complete break with previous policy. The World Bank now perceives telecommunications as a major factor in stimulating economic growth with ramifications in areas such as health care and education. In developed countries, a definite obstacle to the widespread introduction of Internet based facilities is the inevitable resistance to change from the suppliers of existing telecommunication and cable television networks wishing to capitalize on their present infrastructure. In developing countries, a lack of telecommunication and cable television infrastructure has thus been seen as a distinct advantage.

Above we have touched upon the open nature of the Internet in the elaboration of its standards. This means that participation is open and available to anyone without the expense of travel and independent of distance. The WWW offers possibilities for publication. With Internet connection, scientists from developing countries can return to their home lands and nevertheless stay in contact with other scientists across the globe.

## Footnotes

1 W3VL: Crystallography has now taken the name “Crystallography World Wide” and is distributed from five mirror sites in Geneva, Johannesburg, Paris, San Diego, and Tokyo.
